# Digital Mental Health: The Answer to the Global Mental Health Crisis?

**DOI:** 10.2196/18472

**Published:** 2020-06-02

**Authors:** Brittany N Rudd, Rinad S Beidas

**Affiliations:** 1 Institute for Juvenile Research Department of Psychiatry University of Illinois at Chicago Chicago, IL United States; 2 Center for Mental Health, Department of Psychiatry Perelman School of Medicine University of Pennsylvania Philadelphia, PA United States; 3 Penn Implementation Science Center, Leonard Davis Institute of Health Economics, University of Pennsylvania Philadelphia, PA United States; 4 Department of Medical Ethics and Health Policy Perelman School of Medicine University of Pennsylvania Philadelphia, PA United States; 5 Department of Medicine Perelman School of Medicine University of Pennsylvania Philadelphia, PA United States

**Keywords:** public mental health, universal mental health prevention, digital implementation support

## Abstract

Digital mental health interventions are often touted as the solution to the global mental health crisis. However, moving mental health care from the hands of professionals and into digital apps may further isolate individuals who need human connection the most. In this commentary, we argue that people, our society’s greatest resource, are as ubiquitous as technology. Thus, we argue that research focused on using technology to support all people in delivering mental health prevention and intervention deserves greater attention in the coming decade.

## Introduction

Mental health is public health. Depression is a leading cause of disability worldwide and is estimated to affect 264 million people globally [1]. Every day 129 Americans die by suicide and 130 die by opioid overdose [2,3]. For nearly every leading cause of death (eg, cancer, heart disease), comorbid mental health disorders increase morbidity and mortality rates. For example, heart disease is the leading cause of death in the United States. Depression increases the risk for coronary heart disease among healthy adults [4,5] and mortality among patients with coronary heart disease [5,6]. To address global public health, we must address mental health.

A comprehensive public health response to disease elimination requires the use of three interrelated services that span prevention and intervention. These three services include universal prevention to limit the development of the disease, targeted intervention among those at greatest risk for acquiring the disease, and intensive intervention to treat those already experiencing the disease. The dominant paradigm for promoting mental health in society is the provision of clinical services to individuals in specialized mental health care facilities, which represents an intensive public health intervention. However, most people in the world with a mental health disorder receive no treatment [7]. Among people who actually receive care, few receive an effective treatment. For example, only 16.5% of people with major depressive disorder receive minimally adequate treatment globally [8]. Although the causes of this treatment-use gap are multifactorial, it is partly due to the dramatic shortage of specialty mental health care providers. The supply of mental health professionals does not meet the demand for mental health care. By 2025, the United States is projected to have shortages in nearly every type of mental health care provider (eg, psychiatrist, psychologist, social worker) [9]. Mental health workforce shortages are even more profound in low- and middle-income countries. The median number of psychiatrists available per 100,000 individuals in high-income countries is 172 times greater than in low-income countries, where there is approximately 0.05 psychiatrists per 100,000 individuals [10]. We require novel models of intervention delivery [11] as well as a paradigm shift toward a comprehensive public mental health response [12] if we are to overcome this global mental health crisis.

A total of 90% of American adults use the internet [13] and 96% own a cellphone [14]. The global penetration rate of the internet is 53%, and although the lowest rates are among Central Africa and Southern Asia, these regions are also experiencing the fastest growth [15]. Widely celebrated as the solution to the supply and demand imbalance in mental health care, digital mental health interventions have flooded the market place to supplement specialty mental health care. However, the evidence supporting their efficacy is mixed [16,17], and engagement with digital mental health interventions, particularly mobile apps that lack ancillary human interaction, is abysmal [18]. Users are unlikely to use these interventions more than a few times. Particularly concerning is the fact that some digital mental health apps share user data without disclosing this in their privacy policy [19]. Furthermore, the movement of specialty mental health care, an intensive public health intervention, from the hands of clinicians and into standalone digital interventions ignores decades of research about the importance of social support and may further isolate individuals who need human connection the most. Given the robust social support literature, it is not surprising that digital interventions with the highest levels of engagement are those that include some form of human interaction [20,21]. In this commentary, we argue for a renewed focus on universal mental health prevention and targeted intervention [22]. We propose that *people*, our society’s greatest resource, are as ubiquitous as technology. Thus, research focused on using technology to support *all people* in their delivery of universal prevention and targeted intervention deserves greater attention in the coming decade. We believe that it is the synergy of people and technology that will allow us to overcome the global mental health crisis, not technology alone.

## The Way Forward

To date, multiple systematic reviews and meta-analytic studies demonstrate that lay health workforces (ie, community members without advanced mental health care training) can deliver effective mental health care, including effectively screening for mental health disorders and providing psychosocial treatments for the most common psychiatric disorders [23,24]. Given the evidence supporting the use of community members in the delivery of targeted and intensive mental health interventions, we envision enabling community members to deliver all forms of public mental health interventions. This includes supporting all people in the delivery of universal interventions through their provision of social support, as well as supporting the delivery of targeted interventions. Specifically, lay individuals in the community can help identify when their loved ones or fellow community members are distressed or in crisis, provide that identified person with evidence-based support, and escalate that person to appropriate levels of care when it is needed. Shifting the work of mental health promotion to all people is the kind of universal public mental health strategy needed to move the needle [22] and attenuate the stigma associated with mental health disorders. Our efforts need to parallel our efforts to solve other public health crises. We need mental health prevention and intervention to be like fluoride in the water—it needs to be everywhere. Given the ubiquity of people, the synergy of people empowered by technology may be the answer that provides a solution to our global mental health crisis.

To advance these efforts, we make the following suggestions:

First, all people need to be able to reliably identify when their loved one or fellow community member is in need of help. There are both active and passive ways to accomplish this goal. An active strategy may involve the dissemination of online trainings that teach all people to recognize the difference between stress, distress, and crisis, aligned with popular gatekeeper trainings [25]. However, there are also more passive strategies that harness technology. Work is underway to use data naturally available in text messages and social media to reliably predict mood states [26], including Facebook’s work to develop an algorithm to predict and prevent suicide among its users [27,28]. These innovative models could eventually support people in identifying mood states in their loved ones. Perhaps in the future our cell phones can notify us when our loved one is engaging in a text message conversation reflective of an urge to use opioids.

Second, we need to distill the active ingredients of our most robust evidence-based practices into core strategies that are ready for dissemination to the public. Identification of problems must be tied to actionable and clear steps and interventions. Thus, as we develop ways for all people to identify stress, distress, and crisis in their loved ones and community members, each category must be connected to evidence-based interventions, including when to connect that person to a higher level of care. To do that, we first need to establish which evidence-based strategies are appropriate for all people to use. Perhaps our cell phones can suggest that we provide our loved one with the National Suicide Prevention Hotline number or call 911 when a loved one is in crisis. When a loved one is stressed or distressed, perhaps our cell phones can coach a friend or family member to use low-intensity evidence-based techniques, such as active listening, problem solving, and behavioral activation as was used in the *Atmiyata* trial among community volunteers to promote wellness and reduce distress in India [29].

Third, we need to identify the best platform for disseminating and implementing the key elements of this proposed public mental health approach. One avenue could be the public education system. Public education curriculum increasingly includes information regarding physical health promotion (eg, health eating and exercise); thus, this may be an appropriate setting for learning about mental health promotion in oneself and others. In addition, as we continue to invest in patient-facing digital interventions to promote mental health, so too should we consider investing in clinician- or support-person-facing digital implementation supports. A notable example of this work includes Naslund, Shidhaye, and Patel’s [30] work to harness digital technology to build capacity for the implementation of evidence-based mental health intervention in nonspecialist health workers. Technology such as this could be adapted and refined so that it can be used to support all people in supporting their loved ones and community members.

Fourth, this work will require attending to several important ethical considerations. Providing mental health support places deliverers at an increased risk for experiencing secondary stress. Encouraging people to ask and provide care might meaningfully increase our population’s experience with secondary stress. Suffering is human, but often people are uncomfortable sitting with the discomfort and suffering of others. Furthermore, this work begs the question of who is responsible for providing support. Is a loved one partially responsible for incidents that occur when they did not respond to their cell phone’s notification? We posit that, for the most part, friends, family, and community members already worry about the health and safety of their friends, family, and community members, and often feel responsible when incidents occur. To overcome this, we want to ensure that everyone has the tools to intervene, rather than unproductively worry, when they are ready and willing. Furthermore, safeguards must be in place to prevent information collected via technology or when a friend intervenes from causing harm (eg, employment discrimination). Thus, privacy concerns must be addressed. For example, one might argue that surveillance data collected to predict high-risk behaviors, like suicidal behavior on Facebook, is protected health information and should, therefore, be subject to regulatory oversight.

To solve the mental health crisis, we need innovative and expansive solutions, and we believe that harnessing the power of people in mental health prevention and intervention delivery, supported by technology, may get us one step closer to improving public mental health.

## References

[1] GBD 2017 Disease and Injury Incidence and Prevalence Collaborators (2018). Global, regional, and national incidence, prevalence, and years lived with disability for 354 diseases and injuries for 195 countries and territories, 1990–2017: a systematic analysis for the Global Burden of Disease Study 2017. Lancet.

[2] (2020). Centers for Disease Control and Prevention.

[3] (2020). American Foundation for Suicide Prevention.

[4] Pan A, Sun Q, Okereke OI, Rexrode KM, Hu FB (2011). Depression and risk of stroke morbidity and mortality: a meta-analysis and systematic review. JAMA.

[5] Shi S, Liu T, Liang J, Hu D, Yang B (2017). Depression and risk of sudden cardiac death and arrhythmias. Psychosom Med.

[6] Barth J, Schumacher M, Herrmann-Lingen C (2004). Depression as a risk factor for mortality in patients with coronary heart disease: a meta-analysis. Psychosom Med.

[7] Wang PS, Aguilar-Gaxiola S, Alonso J, Angermeyer MC, Borges G, Bromet EJ, Bruffaerts R, de Girolamo G, de Graaf R, Gureje O, Haro JM, Karam EG, Kessler RC, Kovess V, Lane MC, Lee S, Levinson D, Ono Y, Petukhova M, Posada-Villa J, Seedat S, Wells JE (2007). Use of mental health services for anxiety, mood, and substance disorders in 17 countries in the WHO world mental health surveys. Lancet.

[8] Thornicroft G, Chatterji S, Evans-Lacko S, Gruber M, Sampson N, Aguilar-Gaxiola S, Al-Hamzawi A, Alonso J, Andrade L, Borges G, Bruffaerts R, Bunting B, de Almeida JMC, Florescu S, de Girolamo G, Gureje O, Haro JM, He Y, Hinkov H, Karam E, Kawakami N, Lee S, Navarro-Mateu F, Piazza M, Posada-Villa J, de Galvis YT, Kessler RC (2017). Undertreatment of people with major depressive disorder in 21 countries. Br J Psychiatry.

[9] Health Resources and Services Administration/National Center for Health Workforce Analysis; Substance Abuse and Mental Health Services Administration/Office of Policy, Planning, and Innovation (2015). National projections of supply and demand for behavioral health practitioners: 2013-2025. HRSA.

[10] Kakuma R, Minas H, van Ginneken N, Dal Poz MR, Desiraju K, Morris JE, Saxena S, Scheffler RM (2011). Human resources for mental health care: current situation and strategies for action. Lancet.

[11] Kazdin AE (2018). Annual research review: expanding mental health services through novel models of intervention delivery. J Child Psychol Psychiatr.

[12] Atkins MS, Frazier SL (2011). Expanding the toolkit or changing the paradigm: are we ready for a public health approach to mental health?. Perspect Psychol Sci.

[13] (2019). Pew Research Center.

[14] (2019). Pew Research Center.

[15] Kemp S (2018). We Are Social.

[16] Weisel K, Fuhrmann L, Berking M, Baumeister H, Cuijpers P, Ebert D (2019). Standalone smartphone apps for mental health-a systematic review and meta-analysis. NPJ Digit Med.

[17] Firth J, Torous J, Nicholas J, Carney R, Rosenbaum S, Sarris J (2017). Can smartphone mental health interventions reduce symptoms of anxiety? A meta-analysis of randomized controlled trials. J Affect Disord.

[18] Torous J, Nicholas J, Larsen ME, Firth J, Christensen H (2018). Clinical review of user engagement with mental health smartphone apps: evidence, theory and improvements. Evid Based Ment Health.

[19] Huckvale K, Torous J, Larsen ME (2019). Assessment of the data sharing and privacy practices of smartphone apps for depression and smoking cessation. JAMA Netw Open.

[20] Kim H, Ray CD, Veluscek AM (2017). Complementary support from facilitators and peers for promoting mhealth engagement and weight loss. J Health Commun.

[21] Piette JD, Marinec N, Janda K, Morgan E, Schantz K, Yujra ACA, Pinto B, Soto JMH, Janevic M, Aikens JE (2016). Structured caregiver feedback enhances engagement and impact of mobile health support: a randomized trial in a lower-middle-income country. Telemed J E Health.

[22] Purtle J, Nelson KL, Counts NZ, Yudell M (2020). Population-based approaches to mental health: history, strategies, and evidence. Annu Rev Public Health.

[23] Barnett ML, Gonzalez A, Miranda J, Chavira DA, Lau AS (2018). Mobilizing community health workers to address mental health disparities for underserved populations: a systematic review. Adm Policy Ment Health.

[24] Singla DR, Kohrt BA, Murray LK, Anand A, Chorpita BF, Patel V (2017). Psychological treatments for the world: lessons from low- and middle-income countries. Annu Rev Clin Psychol.

[25] Burnette C, Ramchand R, Ayer L (2015). Gatekeeper training for suicide prevention: a theoretical model and review of the empirical literature. Rand Health Q.

[26] Zulueta J, Piscitello A, Rasic M, Easter R, Babu P, Langenecker SA, McInnis M, Ajilore O, Nelson PC, Ryan K, Leow A (2018). Predicting mood disturbance severity with mobile phone keystroke metadata: a biaffect digital phenotyping study. J Med Internet Res.

[27] (2020). Facebook.

[28] Tsukayama H (2017). The Washington Post.

[29] Shields-Zeeman L, Pathare S, Walters BH, Kapadia-Kundu N, Joag K (2017). Promoting wellbeing and improving access to mental health care through community champions in rural India: the intervention approach. Int J Ment Health Syst.

[30] Naslund JA, Shidhaye R, Patel V (2019). Digital technology for building capacity of nonspecialist health workers for task sharing and scaling up mental health care globally. Harvard Rev Psychiatry.

